# Hydroxyl Derivatives of Oils from Solid Fats as Components for Production of Polyurethane Foams

**DOI:** 10.3390/molecules30132703

**Published:** 2025-06-23

**Authors:** Elżbieta Malewska, Maria Kurańska, Klara Grelowska, Aleksandra Put, Hubert Ożóg, Julia Sędzimir, Natalia Kowalik, Michał Kucała, Aleksander Prociak

**Affiliations:** 1 Cracow University of Technology, Faculty of Chemical Engineering and Technology, Warszawska 24, 31-155 Cracow, Poland; elzbieta.malewska@pk.edu.pl (E.M.); klara.grelowska@student.pk.edu.pl (K.G.); aleksandra.put@student.pk.edu.pl (A.P.); hubert.ozog@student.pk.edu.pl (H.O.); julia.sedzimir@student.pk.edu.pl (J.S.); natalia.kowalik18@student.pk.edu.pl (N.K.); aleksander.prociak@pk.edu.pl (A.P.); 2 Cracow University of Technology, CUT Doctoral School, Faculty of Chemical Engineering and Technology, Warszawska 24, 31-155 Cracow, Poland; michal.kucala@doktorant.pk.edu.pl

**Keywords:** solid fats, biopolyols, polyurethane foams, European Green Deal

## Abstract

Biopolyols derived from solid fats of both vegetable origin (coconut oil (P/CO) and palm oil (P/PA)) and animal origin (pork fat (P/PO) and duck fat (P/DU)) were used to produce thermal insulation polyurethane foams. The biopolyols were characterized by hydroxyl numbers in the range of 341–396 mgKOH/g, a viscosity of 60–88 mPa·s, and a functionality of 2.3–3.4. Open-cell polyurethane foams were obtained by replacing from 50 to 100 wt.% of a petrochemical polyol with the biopolyols from solid fats. The most advantageous properties were found for the materials modified with the biopolyol based on pork fat, which was attributed to its high degree of cell openness. At a low apparent density, the foam materials were characterized by good dimensional stability. The use of solid fats offers new possibilities for modifying thermal insulation polyurethane foams.

## 1. Introduction

The chemical industry in Europe is striving for a major change. It aims to achieve climate neutrality and a transition to safe and sustainable chemicals by 2050. This is a transformation that requires major investments and the development of solutions that are seen as essential to achieving the ambitious environmental goals. It is key to the implementation of the European Green Deal [1]. In this context, the transition to a carbon-neutral economy has become an urgent task and a significant opportunity to improve the quality of the environment. A key challenge in climate protection is to achieve a balance between greenhouse gas emissions and their sequestration and absorption by ecosystems. Carbon neutrality initiatives include replacing petrochemical raw materials with renewable raw materials [2,3].

Polyurethane (PU) materials can play an important role in achieving this goal. Firstly, rigid PU foams offer excellent thermal insulation, which ensures a reduction in heat loss, thus reducing greenhouse gases emitted into the atmosphere in connection with heating buildings [4]. The second aspect is the reduction in greenhouse gas emissions at the stage of PU foam production by replacing petrochemical raw materials with renewable ones, including waste. The most frequently used renewable raw materials for the production of polyols are vegetable oils. The preparation of biopolyols that are based on different types of oils, such as canola oil, sunflower oil, and palm and rapeseed oil, has already been reported [5]. Depending on their chemical structure, oils can be liquid or solid fats. One of the solid fats used on a large scale is palm oil. An advantage of palm oil as a source of polyol is that it is abundant and chemically has an unsaturated fatty acid composition. The direct application of vegetable oils, particularly edible oils, in the PU industry is disadvantageous as it directly competes with food. In consequence, there is a need to develop more sustainable initiatives. One such initiative is the use of waste frying oils. Studies have been conducted on the effect of modified frying oils on the properties of PU foams, and it has been shown that they can be a full-value substrate for the synthesis of polyurethanes [6]. Polaczek et al. studied the influence of palm oil’s origin on the properties of biopolyols. Three oil versions were chosen: refined, unrefined, and used cooking palm oil. The study showed that used cooking palm oil may be a substitute for fresh oil. It was also found that a high content of free fatty acids and impurities in unrefined palm oil may lead to the formation of side products, which have an impact on the chemical structures of the biopolyols. However, it was concluded that there was no negative influence of the chemical structure on the properties of the PU foams [7]. Other studies have also been conducted on the use of modified frying oils in the synthesis of open-cell and closed-cell PU foams. It has been found that this type of waste can successfully replace fresh vegetable oil in the synthesis of polyurethanes, which will eliminate the problem of competition for food [8,9,10].

Another type of solid fat, which has not yet been described with regard to the possibility of its use in the modification of low-apparent-density polyurethane foams, is animal fats. The animal by-products production in the European Union amounts to approximately 17 Mt/year. In France, approximately 3 Mt of animal by-products are created each year, of which 300 kt of fat remains after the rendering process per year [8]. One of the methods of managing animal fat waste is the production of biodiesel [11,12,13].

Increasing food production leads to a greater amount of waste. Therefore, the analysis of the possibilities of using hydroxyl derivatives of oils from solid fats as components for the production of PU foams is also of interest from an application point of view. Solid animal oils were nearly not used in PU’s. In this paper, transesterification processes for selected solid fats of both vegetable and animal origin were applied to obtain biopolyols for PU systems. The aim of this work was to analyze the effect of such biopolyols on the cell structure and physical–mechanical properties of PU foams modified with them.

## 2. Results and Discussion

**Figure 1:**
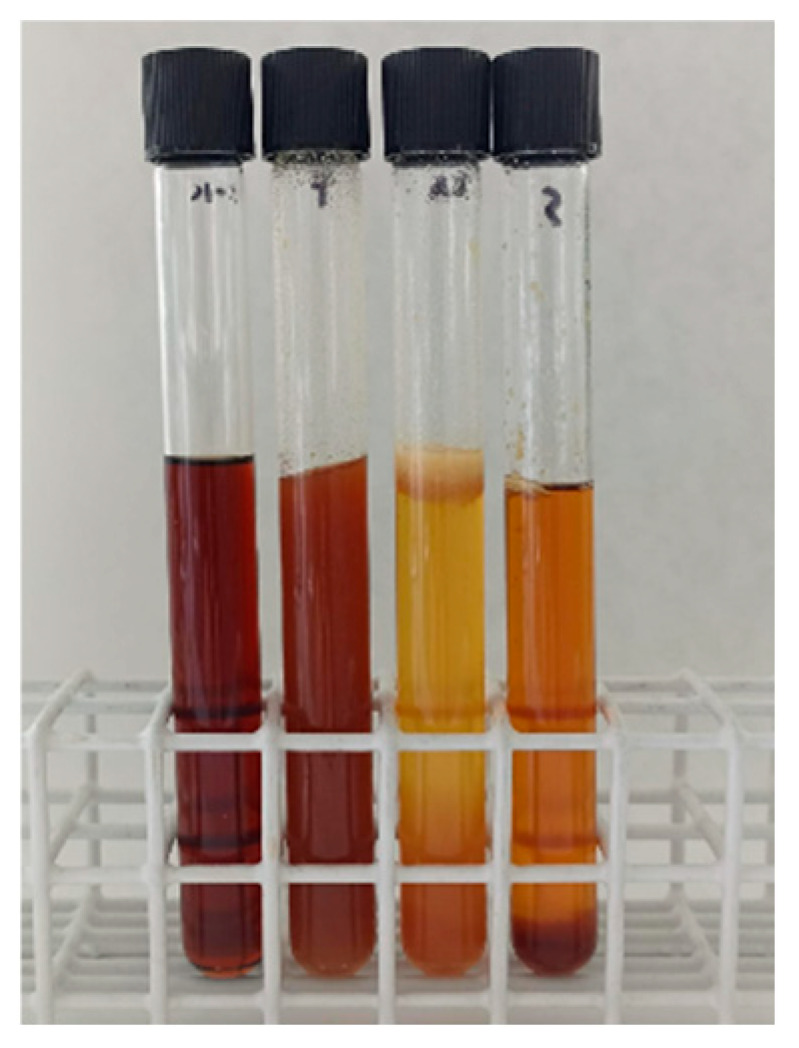
Biopolyols based on coconut oil (P/CO), palm oil (P/PA), duck fat (P/DU), and pork fat (P/PO).

Waste from solid fats can be successfully used to produce biopolyols for the synthesis of plastics. The high degree of saturation of oils means that the reaction that can be used to modify vegetable oils is mainly the transesterification reaction. Table 1 presents the properties of biopolyols based on coconut oil (P/CO), palm oil (P/PA), pork fat (P/PO), and duck fat (P/DU) obtained as a result of transesterification. The biopolyols are shown in Figure 1.

The biopolyols obtained by the transesterification of solid fats with triethanolamine were characterized by hydroxyl number values comparable to those of the biopolyols obtained in the same reaction from other oils, e.g., from rapeseed oil [14]. These values of hydroxyl numbers allow the use of the obtained biopolyols in the synthesis of new PU materials. The viscosity values of the biopolyols were also at a satisfactory level.

**Figure 2:**
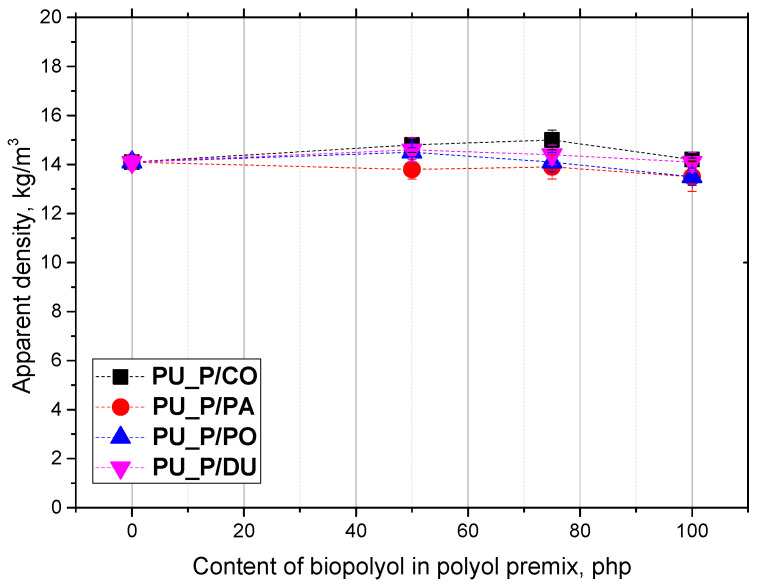
Apparent densities of polyurethane foams.

One of the main quantities of rigid PU foams that determines their mechanical and thermal insulation properties, as well as their dimensional stability, is their apparent density. Figure 2 presents the influence of the type and content of biopolyols on the apparent density of open-cell PU foams.

The apparent densities of the biofoams had similar values and ranged from 13 to 15 kg/m^3^. In the literature, open-cell polyurethane foams obtained from liquid vegetable oils characterized by a high degree of unsaturation have been described. Polaczek and Kurańska obtained biofoams modified with biopolyols based on vegetable oils: hemp seed oil, oil radish oil, rapeseed oil, and used rapeseed oil from the frying process. The open-cell PU foams prepared by them under laboratory conditions were characterized by an apparent density in the range of 11.2 to 12.1 kg/m^3^ [15]. In the case of the foams modified with biopolyols from solid vegetable oils, such as refined palm oil, unrefined palm oil, and used frying palm oil, foams with an apparent density in the range of 13–15 kg/m^3^ were obtained. In those foams, 20% of the petrochemical polyol was replaced with biopolyols. Biopolyols from palm oil were obtained as a result of epoxidation and oxirane ring-opening reactions [7]. The apparent density values of the open-cell PU foams presented in the present work do not differ much from the values described in the literature.

**Figure 3:**
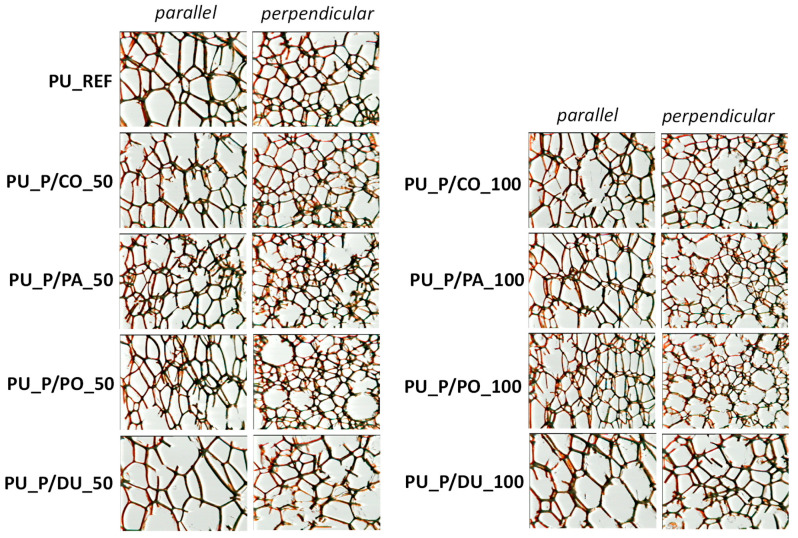
Cellular structure of tested PU biofoams.

**Figure 4:**
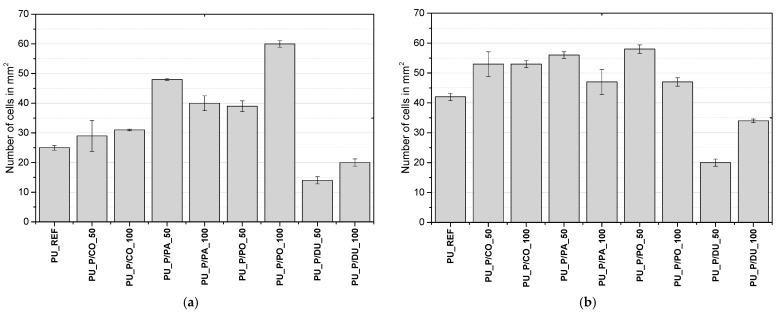
Number of cells in the cross-sections of PU foams per cm^2^ in a direction parallel (**a**) and perpendicular (**b**) to the direction of foam growth.

Optical micrographs of the cross-sections of the foams taken in directions parallel and perpendicular to the growth direction of the PU system are shown in Figure 3. The numbers of cells per cm^2^, their average areas, and their anisotropy coefficients are shown in Figure 4 and Table 2.

**Figure 5:**
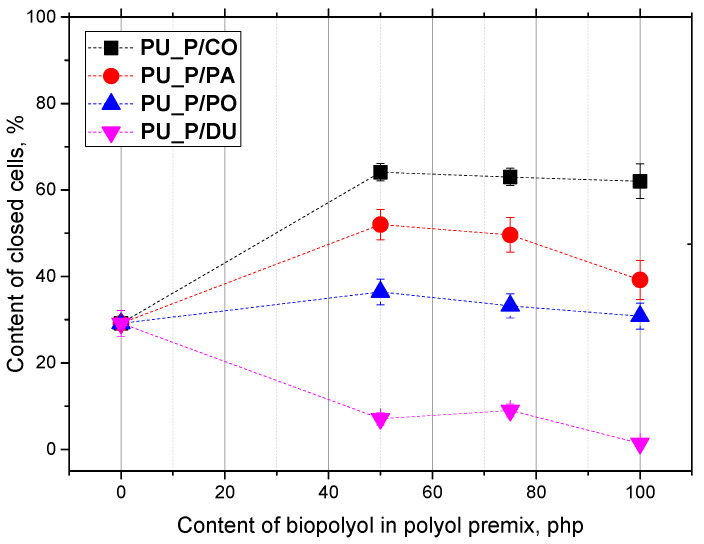
Contents of closed cells in PU biofoams.

The lowest number of cells per cm^2^ and, thus, the largest cross-sectional area of the cells in both the perpendicular and parallel directions were found for the foams derived from the biopolyol based on duck fat. What is more, those materials had the lowest content of closed cells (Figure 5).

This effect may be due to the rapid and large cell growth during the foaming process, which led to cell rupture. Furthermore, the duck-fat biopolyol had a lower viscosity in comparison with the P/PA biopolyol, despite its comparable hydroxyl number.

**Figure 6:**
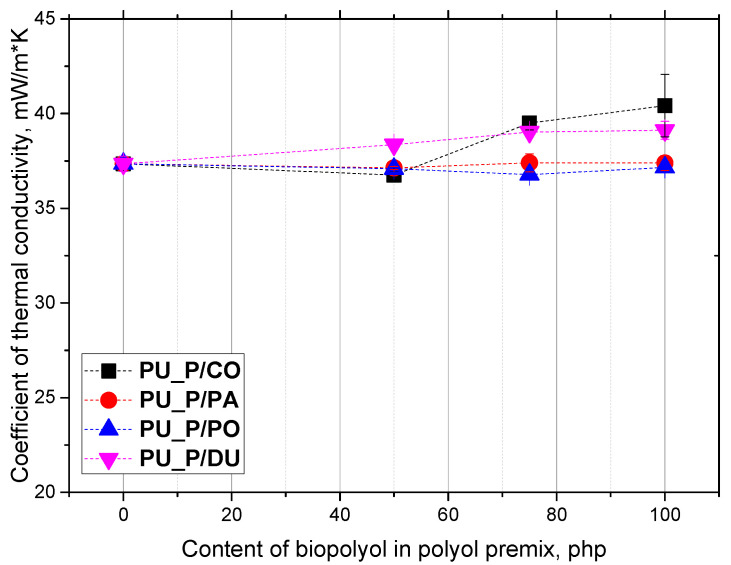
Content of closed cells in PU biofoams vs. biopolyol content in polyol premix.

For low-density PU foams, the closed-cell content is of great importance for dimensional stability. Foams with a cell content above 50% may be dimensionally unstable if their mechanical strength is lower than the atmospheric pressure acting on them. The obtained foams were characterized by an anisotropic structure. The anisotropy coefficient values in the direction parallel to the growth direction of the PU system were greater than one, which indicated the elongation of the cells in the growth direction. In the case of the perpendicular direction, the anisotropy coefficient values were close to one, indicating an isotropic cell shape. Bearing in mind the main application of rigid PUR foams in thermal insulation, it is crucial to maintain the heat transfer coefficient value at the lowest possible level. Figure 6 shows the effect of the content and type of biopolyol on the value of the thermal conductivity coefficient.

The thermal conductivity coefficient values for the foams modified with the biopolyols from solid fats ranged from 0.036 to 0.040 W/m·K. The thermal conductivity coefficient values found in this work did not differ from those given in the literature for other open-cell PUR biofoams. M. Kurańska et al. analyzed foam materials obtained from biopolyols of fruit seed oils in laboratory conditions. Their foams were characterized by a thermal conductivity coefficient in the range from 0.038 to 0.042 W/(m·K) [16].

**Figure 7:**
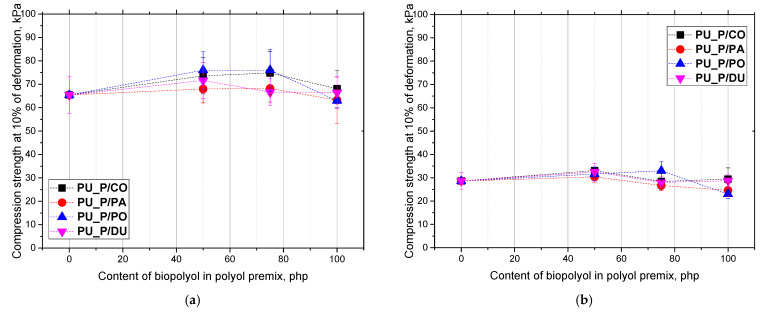
Compression strength (**a**) parallel and (**b**) perpendicular to the growth direction of biofoams vs. biopolyol content in polyol premix.

The mechanical properties of the foams were tested in two directions: parallel and perpendicular to the foam growth direction (Figure 7). Such an approach was motivated by the anisotropy of the cells, which causes different mechanical properties of foams depending on the direction in which they are measured.

The compressive strength values in the parallel direction were higher compared with those measured in the perpendicular direction, which was a consequence of the anisotropic nature of the obtained foams.

The compressive strength at 10% stress of the PU foams reached values of up to 70 kPa in a direction parallel and about 30 kPa in a direction perpendicular to the foam growth direction. These are optimal values as far as the requirements set for commercial PU foams are concerned. The highest values of compressive strength were observed for the PU biofoam modified with the biopolyol from coconut oil, which was probably due to its higher content of closed cells in comparison with the other materials.

Regardless of the used biopolyol, all modified foams achieved strength values much greater than 10 kPa, which is a minimum demand in the case of commercial foams. Moreover, the mechanical properties of the tested biofoams were at a similar or higher level compared with other foams described in the literature. E. Malewska described foam systems modified with biopolyols from radish seed oil. The highest compressive strength in the perpendicular direction was observed for foams obtained using a biopolyol synthesized in a reaction with ethylene glycol (~33 kPa), while the lowest one was for foams made with a biopolyol synthesized in a reaction with triethanolamine (~23 kPa).

The difference between the internal cell gas pressure in the closed-cell foams and the external atmospheric pressure can lead to the dimensional instability of such materials with a low apparent density. However, in the case of foams with a low apparent density and a dominant open-cell structure, there is no pressure difference that could cause dimensional instability. The results of the dimensional stability of the biofoams tested at different temperatures are shown in Table 3.

The obtained foams were characterized by very good dimensional stability. Although the foams containing biopolyols from coconut oil, palm oil, and pork fat were characterized by closed-cell contents higher than 20% and low apparent densities, the dimensional change both at low and elevated temperatures did not exceed 1% in most cases.

## 3. Materials and Methods

### 3.1. Biopolyol Synthesis and Characterization

The characteristics of solid vegetable oils, such as coconut (CO) and palm (PA) oils, as well as those of animal origin, such as pork (PO) and duck fat (DU), are shown in Table 4.

Triethanolamine (TEA) (Avantor Performance Materials Poland, Gliwice, Poland) as a transesterification agent and anhydrous zinc acetate (in an amount of 0.3% by mass) (Chempur, Piekary Śląskie, Poland) as a catalyst in the synthesis of biopolyols were used. Biopolyols were synthesized using the transesterification of selected oils (CO, PA, PO, and DU) with TEA. The synthesis of the biopolyols was carried out at a temperature of 175 °C for 2 h. The molar ratio of oil to TEA was 1:3.

Iodine value (Ival) analysis was performed using the Hanus method according to the PN-87/C-04281 standard [17]. This procedure involves the addition of halogen atoms at the sites of double bonds in unsaturated compounds. The unsaturation degree of the compound is expressed as the mass of iodine that has been added. The hydroxyl values (Hv) of the polyols were determined by titration using the pyridine method in accordance with the PN-93/C-89052/03 standard [18]. In this method, an acetylating reagent was used, which is a solution of acetic anhydride and dimethylformamide mixed in appropriate volume proportions. A catalyst solution resulting from mixing dimethylaminopyridine with dimethylformamide in appropriate proportions was also used. A solution of thymolphthalein in dimethylformamide served as an indicator. The number average molecular weight (Mn) and dispersity (D) of the compounds used in the experiments were determined using a gel permeation chromatograph made by Knauer (Berlin, Germany) based on calibration for polystyrene standards. The device was equipped with a thermostatic device with a Plgel MIXED-E column and a refractometric detector. The analyses were performed at 35 °C. Tetrahydrofuran was used as an eluent, and its flow rate was fixed at 1 mL/min. The functionality (f), related to the number of OH groups per molecule, was calculated according to the following formula: f = (Hv·Mn)/56,110. The viscosity of the biopolyols was measured at 40 °C using a plate–plate rotational viscometer RM 200 CP4000 PLUS by Lamy Rheology (Champagne-au-Mont-d’Or, France), equipped with a PP-20 measuring spindle.

### 3.2. Biofoam Manufacturing and Testing

In the polyurethane systems, the following main components were used: the petrochemical polyol Rokopol^®^ RF-551 (PCC Rokita S.A, Brzeg Dolny, Poland; hydroxyl number: 405 mgKOH/g; viscosity: 3030 mPa·s at 25 °C; water content: 0.1%) and biopolyols (the Department of Chemistry and Technology of Polymers at the Cracow University of Technology, Kraków, Poland). The characteristics of the biopolyols are presented in the Section 2 in Table 2. EKOPUR B (PMDI) was supplied by Minova Ekochem S.A., Siemianowice Śląskie, Poland (isocyanate group content: 31 wt.%; viscosity: 200 mPa·s at 25 °C). The catalysts (POLYCAT^®^ 15, POLYCAT^®^ NP10, and Kosmos 19) and surfactants (TEGOSTAB^®^ B 8526 and Ortegol^®^ 500; Evonik Industries AG, Essen, Germany). The flame-retardant tris(2-chloroisopropyl)phosphate (TCPP), with a viscosity of 67 mPa·s, was supplied by Purinova (Bydgoszcz, Poland). Distilled water was used as a chemical blowing agent. PU foams were prepared using a one-step method with a two-component system. The compositions of the petrochemical polyol and biopolyols, as well as the symbol system for the biofoams, are presented in Table 5. The contents of the catalysts (Polycat 15—0.5 php; Polycat NP10—3.0 php; and Kosmos 19—0.2 php), surfactants (Ortegol 500—0.5 php; Tegostab 8526—5 php), water (20 php), and flame retardant (TCPP-20 php) in all systems were the same.

Component A (polyol premix) consisted of polyols, catalysts, surfactants, a flame retardant, and water, while component B was isocyanate. The polyol premix was mixed for 30 s, component B was added to component A, and the mixture was stirred for approximately 10 s. The reaction mixture was poured into an open vertical mold. The isocyanate index (INCO) of the PUR foams was 1.1. The obtained foams were removed from the mold after 24 h, and then the samples were cut out in order to test them.

The apparent density of the foam materials was calculated as a ratio of the masses and volumes of the samples in accordance with the standard ISO 845 [19]. The cell morphology was analyzed using an optical microscope (PZO, Warszawa, Poland) and the software Aphelion (Version 3.1). The images were taken in two cross-sections to the direction of foam growth: perpendicular and parallel. The thermal conductivity coefficient of the rigid PU foams was analyzed using the Laser Comp Heat Flow Instrument Fox 200 (TA Instruments, New Castle, DE, USA) according to ISO 8301 [20]. During the thermal conductivity measurement, a one-way heat flow between the hot (20 °C) and cold (0 °C) plates was established. Compressive strength tests were carried out using the Zwick Z005 device (Ulm, Germany) according to ISO 844 [21] in the directions parallel and perpendicular to the direction of foam rise. The dimensional stability of the foams was tested according to the ISO 2796-1986 [22] standard at −25 °C and 70 °C.

## 4. Conclusions

The objective of this work was to analyze the effect of biopolyols made from solid vegetable oils and solid animal fats. The foams were prepared by replacing 50, 75, and 100% of a petrochemical polyol with the biopolyols. It was found that the biopolyol (regardless of its type) had a strong effect on the foam cell structure. The largest cross-sectional cell sizes were obtained for the foams modified with the duck fat biopolyol. Moreover, those foams had the lowest closed-cell contents. The polyurethane foams had apparent densities from 13 to 15 kg/m^3^, depending on the content and type of biopolyol. The thermal conductivity of the tested foams ranged between 0.036 and 0.040 W/m·K and is typical for open-cell polymeric foams. The lowest values were obtained for the foams modified with the biopolyols derived from pork fat. The modification with the biopolyols had no negative effect on the dimensional stability of the tested foams. The satisfactory results described in this work suggest that the tested biopolyols based on renewable raw materials may serve as valuable components in the synthesis of polyurethane biofoams, which have the potential to be successfully applied in the production of thermal insulation materials.

## Figures and Tables

**Table 1:** Properties of biopolyols.

Property	P/CO	P/PA	P/PO	P/DU
Hydroxyl value, mgKOH/g	396	341	370	352
Number molecular weight, g/mol	330	510	510	510
Dispersity	1.25	1.27	1.24	1.25
Functionality	2.3	3.1	3.4	3.2
Viscosity, mPa·s	48	88	60	64

**Table 2:** Average cross-sectional area of cells and anisotropy coefficient measured in a direction parallel and perpendicular to the direction of foam growth.

Foam Symbol	Parallel-to-Foam-Growth Direction		Perpendicular-to-Foam-Growth Direction	
	Cross-Sectional Area, ·10^2^ [mm^2^]	Anisotropy Index	Cross-Sectional Area, ·10^2^ [mm^2^]	Anisotropy Index
PU_REF	2.52 · 10^−2^ ± 3.62 · 10^−3^	1.29 ± 8.32 · 10^−2^	9.64 · 10^−3^ ± 8.82 · 10^−3^	1.05 ± 4.73 · 10^−2^
PU_P/CO_50	2.57 · 10^−2^ ± 4.50 · 10^−3^	1.26 ± 1.36 · 10^−1^	1.36 · 10^−2^ ± 1.51 · 10^−3^	1.09 ± 2.28 · 10^−2^
PU_P/CO_100	2.25 · 10^−2^ ± 1.56 · 10^−3^	1.46 ± 3.83 · 10^−2^	1.30 · 10^−2^ ± 9.96 · 10^−4^	0.96 ± 3.01 · 10^−2^
PU_P/PA_50	1.27 · 10^−2^ ± 9.37 · 10^−4^	1.19 ± 1.10 · 10^−1^	1.18 · 10^−2^ ± 1.17 · 10^−3^	1.03 ± 3.98 · 10^−2^
PU_P/PA_100	1.59 · 10^−2^ ± 2.02 · 10^−3^	1.36 ± 1.07 · 10^−1^	1.48 · 10^−2^ ± 8.33 · 10^−4^	1.00 ± 1.69 · 10^−2^
PU_P/PO_50	1.66 · 10^−2^ ± 2.44 · 10^−3^	1.62 ± 1.26 · 10^−1^	1.26 · 10^−2^ ± 6.04 · 10^−4^	0.95 ± 3.33 · 10^−2^
PU_P/PO_100	1.13 · 10^−2^ ± 4.26 · 10^−4^	1.52 ± 3.74 · 10^−2^	1.54 · 10^−2^ ± 6.12 · 10^−4^	1.03 ± 3.77 · 10^−2^
PU_P/DU_50	4.89 · 10^−2^ ± 2.62 · 10^−3^	1.38 ± 8.46 · 10^−2^	3.46 · 10^−2^ ± 5.60 · 10^−3^	1.01 ± 2.48 · 10^−2^
PU_P/DU_100	3.33 · 10^−2^ ± 9.92 · 10^−4^	1.20 ± 6.16 · 10^−2^	1.94 · 10^−2^ ± 1.05 · 10^−3^	0.95 ± 3.65 · 10^−2^

**Table 3:** Dimensional stability of foams measured at 70 °C and −25 °C.

Symbols of Samples	−25 °C			70 °C		
	Length	Width	Thickness	Length	Width	Thickness
REF	0.32	0.28	0.71	0.33	0.18	0.35
PU_P/CO_50	0.25	0.54	0.56	0.02	0.54	−0.12
PU_P/CO_75	0.97	0.65	0.35	0.44	0.27	0.13
PU_P/CO_100	0.49	0.94	0.62	0.18	0.41	0.17
PU_P/PA_50	0.23	0.38	1.04	0.42	0.42	−0.43
PU_P/PA_75	0.08	0.30	0.89	0.23	0.29	−0.33
PU_P/PA_100	0.13	0.30	0.96	0.13	0.26	−0.21
PU_P/PO_50	0.06	0.27	0.62	0.61	0.40	0.15
PU_P/PO_75	0.10	0.12	0.70	0.55	0.39	−0.14
PU_P/PO_100	0.10	0.17	0.58	0.15	0.20	0.13
PU_P/DU_50	0.15	0.14	0.22	0.19	0.21	0.07
PU_P/DU_75	0.09	0.17	0.59	0.08	0.07	0.16
PU_P/DU_100	0.06	0.18	0.40	0.03	−0.10	0.21

**Table 4:** Properties of vegetable oil and animal fats.

Properties	CO	PA	PO	DU
Iodine value, gI2/100 g	10.3	47.5	54.8	64.8
Number average molecular weight, g/mol	748	927	853	929
Dispersity	1.01	1.04	1.01	1.01
Viscosity, mPa·s	19	47	32	28

**Table 5:** Compositions of polyols in polyurethane systems and PU foam symbol system.

Foam Symbol/ Polyol	RF551	P/CO	P/PA	P/PO	P/DU
REF	100	0	0	0	0
PU_P/CO_50	50	50	0	0	0
PU_P/CO_75	25	75	0	0	0
PU_P/CO_100	0	100	0	0	0
PU_P/PA_50	50	0	50	0	0
PU_P/PA_75	25	0	75	0	0
PU_P/PA_100	0	0	100	0	0
PU_P/PO_50	50	0	0	50	0
PU_P/PO_75	25	0	0	75	0
PU_P/PO_100	0	0	0	100	0
PU_P/DU_50	50	0	0	0	50
PU_P/DU_75	25	0	0	0	75
PU_P/DU_100	0	0	0	0	100

## Data Availability

The original contributions presented in this study are included in this article. Further inquiries can be directed to the corresponding author.

## References

[1] Borowicz M., Paciorek-Sadowska J., Isbrandt M. (2025). “From seed to product”—Synthesis of high-value bio-polyol raw materials from white mustard seed processing and assessment of the influence of the degree of oxidation on the properties of the obtained products. Ind. Crops Prod..

[2] Da Silva Hyldmo H., Rye S.A., Vela-Almeida D. (2024). A globally just and inclusive transition? Questioning policy representations of the European Green Deal. Glob. Environ. Change.

[3] Wolf S., Teitge J., Mielke J., Schütze F., Jaeger C. (2021). The European Green Deal—More Than Climate Neutrality. Intereconomics.

[4] Paciorek-sadowska J., Borowicz M., Isbrandt M. (2021). Effect of evening primrose (Oenothera biennis) oil cake on the properties of polyurethane/polyisocyanurate bio-composites. Int. J. Mol. Sci..

[5] Członka S., Strakowska A., Kairyte A. (2020). Application of walnut shells-derived biopolyol in the synthesis of rigid polyurethane foams. Materials.

[6] Orjuela A., Clark J. (2020). ScienceDirect Green chemicals from used cooking oils: Trends, challenges, and opportunities. Curr. Opin. Green Sustain. Chem..

[7] Polaczek K., Kurańska M., Auguścik-Królikowska M., Prociak A., Ryszkowska J. (2021). Open-cell polyurethane foams of very low density modified with various palm oil-based bio-polyols in accordance with cleaner production. J. Clean. Prod..

[8] Cappello M., Filippi S., Rossi D., Cinelli P., Anguillesi I., Camodeca C., Orlandini E., Polacco G., Seggiani M. (2024). Waste-Cooking-Oil-Derived Polyols to Produce New Sustainable Rigid Polyurethane Foams. Sustainability.

[9] Sarim M., Nikje M., Dargahi M. (2023). Preparation and Characterization of Polyurethane Rigid FoamNanocomposites from Used Cooking Oil and Perlite. Int. J. Polym. Sci..

[10] Kurańska M., Polaczek K., Auguścik-Królikowska M., Prociak A., Ryszkowska J. (2020). Open-cell rigid polyurethane bio-foams based on modified used cooking oil. Polymer.

[11] Hasan N., Ratnam M.V. (2022). Biodiesel Production from Waste Animal Fat by Transesterification Using H_2_SO_4_ and KOH Catalysts: A Study of Physiochemical Properties. Int. J. Chem. Eng..

[12] Andreo-Martínez P., Ortiz-Martínez V.M., Salar-García M.J., Veiga-del-Baño J.M., Chica A., Quesada-Medina J. (2022). Waste animal fats as feedstock for biodiesel production using non-catalytic supercritical alcohol transesterification: A perspective by the PRISMA methodology. Energy Sustain. Dev..

[13] Refaat A.A. (2009). Correlation between the chemical structure of biodiesel and its physical properties. Int. J. Environ. Sci. Tech..

[14] Stirna U., Fridrihsone A., Lazdin B., Misane M., Vilsone D. (2013). Biobased Polyurethanes from Rapeseed Oil Polyols: Structure, Mechanical and Thermal Properties. J. Polym. Environ..

[15] Polaczek K., Kurańska M. (2022). Hemp Seed Oil and Oilseed Radish Oil as New Sources of Raw Materials for the Synthesis of Bio-Polyols for Open-Cell Polyurethane Foams. Materials.

[16] Malewska E., Kurańska M., Tenczyńska M., Prociak A. (2024). Application of Modified Seed Oils of Selected Fruits in the Synthesis of Polyurethane Thermal Insulating Materials. Materials.

[17] (1981). Determination of Iodine Value.

[18] (1993). Polyethers for Polyurethanes—Test Methods—Determination of the Hydroxyl Value.

[19] (2006). Cellular Plastics and Rubbers—Determination of Apparent Density.

[20] (1991). Thermal Insulation—Determination of Steady-State Thermal Resistance and Related Properties—Heat Flow Meter Apparatus.

[21] (2021). Rigid Cellular Plastics—Determination of Compression Properties.

[22] (1986). Cellular Plastics, Rigid—Test for Dimensional Stability.

